# Systemic inflammation and family history in relation to the prevalence of type 2 diabetes based on an alternating decision tree

**DOI:** 10.1038/srep45502

**Published:** 2017-03-31

**Authors:** Hirokazu Uemura, A. Ammar Ghaibeh, Sakurako Katsuura-Kamano, Miwa Yamaguchi, Tirani Bahari, Masashi Ishizu, Hiroki Moriguchi, Kokichi Arisawa

**Affiliations:** 1 Department of Preventive Medicine, Institute of Biomedical Sciences, Tokushima University Graduate School, 3-18-15, Kuramoto-cho, Tokushima 770-8503, Japan; 2 Department of Medical Informatics, Institute of Biomedical Sciences, Tokushima University Graduate School, 3-18-15, Kuramoto-cho, Tokushima 770-8503, Japan

## Abstract

To investigate unknown patterns associated with type 2 diabetes in the Japanese population, we first used an alternating decision tree (ADTree) algorithm, a powerful classification algorithm from data mining, for the data from 1,102 subjects aged 35–69 years. On the basis of the investigated patterns, we then evaluated the associations of serum high-sensitivity C-reactive protein (hs-CRP) as a biomarker of systemic inflammation and family history of diabetes (negative, positive or unknown) with the prevalence of type 2 diabetes because their detailed associations have been scarcely reported. Elevated serum hs-CRP levels were proportionally associated with the increased prevalence of type 2 diabetes after adjusting for probable covariates, including body mass index and family history of diabetes (*P* for trend = 0.016). Stratified analyses revealed that elevated serum hs-CRP levels were proportionally associated with increased prevalence of diabetes in subjects without a family history of diabetes (*P* for trend = 0.020) but not in those with a family history or with an unknown family history of diabetes. Our study demonstrates that systemic inflammation was proportionally associated with increased prevalence of type 2 diabetes even after adjusting for body mass index, especially in subjects without a family history of diabetes.

The prevalence of type 2 diabetes has been increasing globally^1^. Obesity is a key risk factor for various chronic diseases, including type 2 diabetes^2^,^3^. A number of other risk factors have also been identified for type 2 diabetes. However, unknown factors or patterns may also affect the risk for type 2 diabetes. We have conducted a prospective cohort study since 2008 in Tokushima Prefecture, Japan. This cohort study has included considerable baseline data (about 240 variables) on medical history, family history of diseases, lifestyle characteristics, anthropometric measurements, and biochemical examinations^4^,^5^.

In the present study, we first generated a prediction model for the prevalence of type 2 diabetes in this baseline dataset using alternating decision tree (ADTree) algorithm^6^, a powerful classification algorithm for data mining. There has been a recent increase in the use of data mining methods in various aspects of medicine due to their promising results^7^,^8^,^9^. Data mining aims to extract useful information from available data by applying techniques from various fields including databases, machine learning, statistics, and visualization^10^.

The resulting ADTree in the present study indicated that having a positive or unknown family history of diabetes showed positive impact on the prevalence of type 2 diabetes; furthermore, in subjects without a family history of diabetes, increased serum levels (≥0.26 mg/L) of high-sensitivity C-reactive protein (hs-CRP), a systemic inflammatory marker, showed positive impact on the prevalence of type 2 diabetes. Having a family history of diabetes is a well-recognized risk factor for developing type 2 diabetes^11^. Low-grade systemic inflammation has also been reported to be associated with various diseases including type 2 diabetes^12^,^13^. However, the combined associations of family history of diabetes and systemic inflammation with type 2 diabetes have been scarcely reported, especially in the Japanese population. Therefore, we performed hypothesis testing and logistic regression analyses in order to calculate the statistical significance and odds ratios of the factors identified by the ADTree.

## Methods

### Study subjects

This cross-sectional study included participants aged 35–69 years who participated in the baseline survey of a prospective cohort study from January 2008 to February 2013 in Tokushima Prefecture, Japan. This study was performed as part of the Japan Multi-Institutional Collaborative Cohort (J-MICC) Study. Details of this cohort study have been reported elsewhere^14^. In brief, the J-MICC Study aims to examine the associations of lifestyle and genetic factors, as well as their interactions with lifestyle-related diseases.

The population in the present study consisted of two groups. The first group consisted of 570 participants who received health examinations at the Tokushima Prefectural General Health Check-up Center from January 2008 to November 2011. The second group consisted of 696 participants who were general inhabitants of Tokushima city and neighboring towns and attended the health check-ups performed by our research team between July 2012 and February 2013. Of the 1,266 participants (637 men and 629 women), 164 (81 men and 83 women) were excluded for the following reasons (overlapping): (1) previous history of ischemic heart disease (n = 29) and/or stroke (n = 14); (2) missing data regarding fasting blood glucose (n = 9), diabetes treatment (n = 0), family history of diabetes (n = 14), or any potential confounding factors included in the multivariable-adjusted models (n = 31); (3) extremely high (>4,000 kcal/day) or low (<1,000 kcal/day) estimated daily energy intake (n = 11); and 4) missing serum hs-CRP values (n = 5) or values ≥ 10 mg/L (n = 15) (confirmed acute inflammatory status), history of rheumatoid arthritis (n = 4), which is accompanied by systemic inflammation, and/or regular use of anti-inflammatory analgesics (n = 48) that could affect serum hs-CRP values. The remaining 1,102 subjects (556 men and 546 women) were included in the analyses.

All participants in the J-MICC Study provided written informed consent prior to participation. This study was conducted according to the principles of the Declaration of Helsinki, and the ethics committees of Nagoya University School of Medicine (the affiliation of the former principal investigator, Nobuyuki Hamajima), Aichi Cancer Center (the affiliation of the current principal investigator, Hideo Tanaka), and Tokushima University Graduate School all approved the protocol of the present study.

### Questionnaire

All participants were requested to complete a structured self-administered questionnaire regarding lifestyle characteristics, including leisure-time exercise, smoking status, alcohol consumption, and dietary habits over the past year at the baseline survey, as described previously^4^,^5^,^15^. This questionnaire also included the questions about medical history and family history of diseases.

Leisure-time exercise was divided into three categories: light exercise such as walking or hiking, moderate exercise such as light jogging or swimming, and vigorous exercise such as marathon running or competitive sports, based on the intensity of the exercise: 3.4, 7.0, and 10.0 metabolic equivalents (METs), respectively. The amount of each exercise category was calculated by multiplying the frequency and duration of each exercise activity (MET level × hours per activity × activity frequency per week); these were summed to estimate the degree of leisure-time exercise and are expressed as MET-hours/week, as described previously^15^.

**Figure 1:**
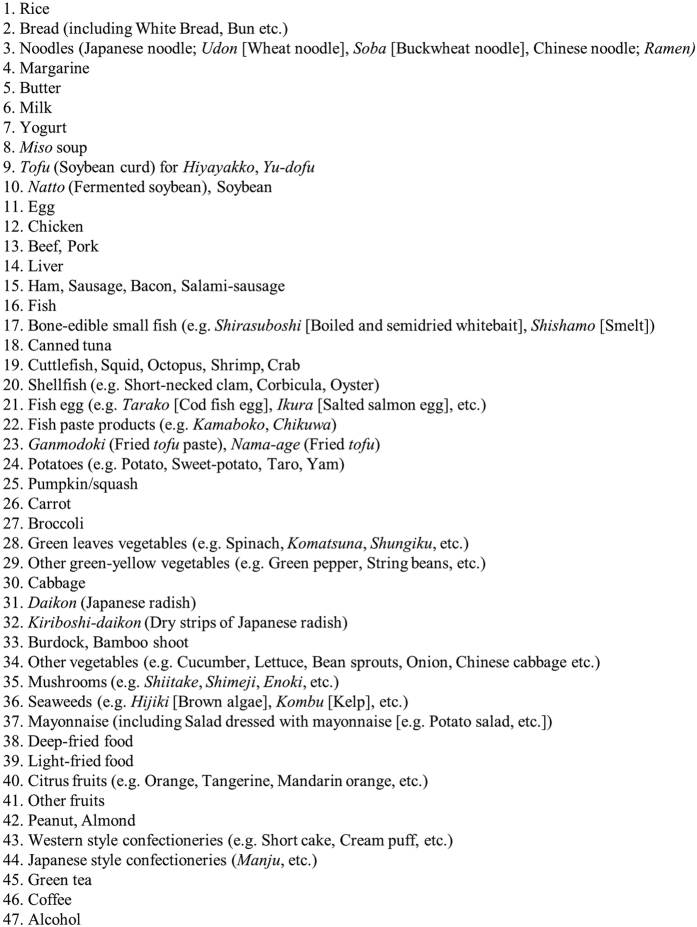
List of the foods and beverages included in the food frequency questionnaire (FFQ).

Dietary evaluations were conducted using a validated short food frequency questionnaire (FFQ)^16^,^17^,^18^,^19^. The FFQ included questions regarding the intake of 47 foods and beverages, listed in Fig. 1, over the previous year. Information concerning the intake frequency and amounts of the 3 staple foods (i.e., rice, bread, and noodles) consumed at breakfast, lunch, and dinner was obtained. The volume and frequency of the consumption of alcoholic beverages including sake, beer, shochu (a Japanese distilled beverage), chuhai (a sweetened beverage mixed with shochu), whiskey, and wine, were determined. Only the intake frequency was obtained for the other 43 foods and beverages as follows (eight categories): three or more times/day (3/day), twice/day (2/day), once/day (1/day), 5–6 times/week (0.8/day), 3–4 times/week (0.5/day), 1–2 times/week (0.2/day), 1–3 times/month (0.1/day), and never or seldom (0/day). Daily energy intake and carbohydrate intake were calculated using a program developed by the Department of Public Health, Nagoya City University School of Medicine^16^,^17^.

Additionally, medical history of diabetes (yes, no, or unknown) for each subject’s mother and father was obtained. When neither the mother nor father had a medical history of diabetes, the subjects was considered negative for a family history of diabetes; when either the mother or father had a medical history of diabetes, the subjects was considered positive for a family history of diabetes. In others cases, a family history of diabetes was regarded as unknown.

### Measurements and diabetes assessment

Body height and weight were measured to the nearest 0.1 cm and 0.1 kg, respectively, at the time of health check-up. Body mass index (BMI) was calculated as weight (kg) divided by height (m) squared. Venous blood was drawn from each participant, and serum was separated within three hours. Fasting plasma glucose levels were obtained from the health check-up data, and hs-CRP levels in stored sera at −80 °C were also measured at an external laboratory (BML Inc., Tokyo, Japan).

Diabetes was defined as a fasting plasma glucose level ≥126 mg/dL or as receiving diabetes treatment^20^.

### Statistical analyses

**Figure 2:**
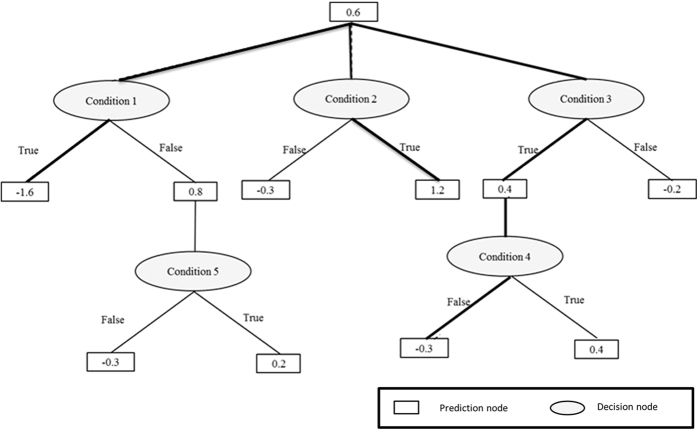
An alternating decision tree (ADTree) structure and prediction method. A case with the following conditions (Condition 1 = True, Condition 2 = True, Condition 3 = True, Condition 4 = False, Condition 5 = True) will be associated with the multi-path with bold solid line. Its diagnosis prediction = sign (0.6 + 0.4–0.3 + 1.2–1.6) = sign (+0.3). This positive sign (+0.3) means positive association with a setting outcome.

**Figure 3:**
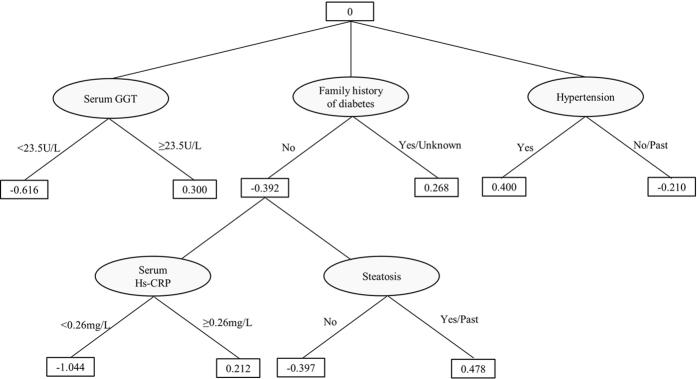
Result of the alternating decision tree (ADTree) for the prevalence of type 2 diabetes. The ADTree indicates that having a positive or unknown family history of diabetes has positive impact and not having a family history has negative impact on the prevalence of type 2 diabetes. In addition, within the group of subjects without a family history of diabetes, elevated serum hs-CRP (≥0.26 mg/L) levels have positive impact on the prevalence of type 2 diabetes, while serum hs-CRP levels <0.26 mg/L have negative impact. GGT, gamma-glutamyl transferase; hs-CRP, high-sensitivity C-reactive protein.

Our data had a large imbalance; i.e. there were a fewer number of cases of type 2 diabetes compared to the number of non-cases. In such situations, standard data mining techniques such as decision trees usually fail to provide good results^21^,^22^. Setoguchi *et al*.^23^ used alternating decision trees in a highly skewed data set to predict the potential for developing pressure ulcers in in-hospital patients. We used a similar approach in our work. Decision trees are powerful classification methods that have been used successfully in many medical studies^24^ because they provide easily understandable graphical classification rules with good accuracy. However, decision trees and many other machine learning and statistical methods encounter difficulties when working with highly skewed and imbalanced data^21^. One solution is to apply a boosting algorithm^25^. Although boosting can improve the performance of decision trees, it results in multiple trees and makes the results difficult to understand. Freund *et al*.^6^ have developed an easy-to-understand decision tree that combines two-layer decision trees (decision stumps) and Adaptive Boosting (AdaBoost)^26^ to generate a single, easily understandable tree called ADTree. An ADTree consists of two kinds of nodes: decision nodes and prediction nodes, as shown in Fig. 2. It starts with a prediction node at its root, then alternates between decision nodes and prediction nodes until it finally terminates with prediction nodes. A decision node provides an inequality condition for one of the input factors (e.g. family history of diabetes) and followed by two prediction nodes correspond to each of the two possible inequality results (True or False). Each prediction node has a real value that indicates the contribution of the corresponding inequality result to the final classification (i.e. diagnosis). Depending on the inequality result further decision nodes might be added before we finally reach a terminal prediction node. The route from the root node to any of the terminal prediction nodes is usually referred to as a *path* where in general an instance can traverse multiple paths. The final classification is determined by the sign of the summation of the values of all prediction nodes that belong to all instance multiple paths. A positive sign indicates a positive class, and a negative sign indicates a negative class. The factors used in the decision node inequality are determined through a search procedure that look for the factor with best classification power. The ADTree was generated using RapidMiner Studio Ver. 7.2 (http://www.rapidminer.com), and was validated using 10-folds cross validation technique^10^. 10-folds cross validation is a low variance estimation method widely used in data mining. The data set is divided into 10 subsets and the classification model is generated 10 times. During each run, one of the subsets is used for validation and the remaining 9 subsets combined are used for model generation. The average and standard deviation of the 10 validations are reported. The generated ADTree’s accuracy, sensitivity, and specificity were 69.6 ± 5.2%, 72.3 ± 22.4%, and 69.4 ± 6.2%, respectively. The ADTree showed that having a positive or an unknown family history of diabetes was related to increased prevalence of type 2 diabetes; within the group of subjects without a family history of diabetes, elevated serum hs-CRP level was related to increased prevalence of type 2 diabetes (shown in Fig. 3). To calculate the statistical importance of each factor in the ADTree, we performed logistic regression analyses. In these analyses, family history of diabetes was divided into two categories according to the ADTree; “negative” and “positive or unknown”. The prevalence rates of diabetes according to “negative”, “positive”, and “unknown” status were 3.2%, 9.2%, and 8.3%, respectively.

Continuous variables were expressed as means ± SD or medians (25^th^ percentile, 75^th^ percentile). Categorical variables were expressed as the number (%). Two sample *t*-test, Wilcoxon rank sum test, or chi-square tests were used to compare the baseline characteristics between subjects having negative and positive/unknown family history of diabetes as appropriate. Logistic regression analyses were performed to evaluate the associations between serum hs-CRP levels and the prevalence of type 2 diabetes after adjusting for the following covariates: (1) age (continuous) and sex (model 1); (2) age, sex, recruitment group (binary), smoking status (current, past, and never), current alcohol drinking (no, yes), leisure-time exercise (MET-hours/week; quartiles), daily carbohydrate intake (g/day; continuous), and daily energy intake (kcal/day; continuous) (model 2); (3) the covariates in model 2 plus BMI (kg/m^2^; quartiles) (model 3); and (4) the covariates in model 3 plus family history of diabetes (negative; positive or unknown) (model 4).

We also evaluated the combined associations of serum hs-CRP level (≤median, >median) and family history of diabetes (negative, positive or unknown) with the prevalence of diabetes by similar logistic regression analyses. The effects of the interactions between serum hs-CRP level and family history of diabetes on the prevalence of diabetes were evaluated by including interaction terms in the models. We further evaluated the associations between serum hs-CRP level (three categories: first plus second quartiles, third quartile, and fourth quartile in all subjects) and the prevalence of diabetes stratified by family history of diabetes. In this stratified analysis, in addition to the strata of negative and positive/unknown family history, the stratum of positive family history alone (excluding unknown family history) was also evaluated, and stratum-specific quartiles of leisure-time exercise and BMI were included in the models.

All calculations and statistical tests were performed using SAS version 9.4 (SAS Institute Inc., Cary, NC, USA). All statistical tests were based on 2-sided probabilities, with a significance level of *P* < 0.05.

## Results

As shown in the resulting ADTree in Fig. 3, having a positive or unknown family history of diabetes showed positive impact on the prevalence of type 2 diabetes; in addition, within the group of subjects without a family history of diabetes, elevated serum hs-CRP levels (≥0.26 mg/L) showed positive impact on the prevalence of type 2 diabetes.

### Baseline characteristics of the subjects according to family history of diabetes

Table 1 shows the baseline characteristics of the subjects according to their family history of diabetes. The prevalence of diabetes was significantly higher (8.8%) in subjects positive for or with an unknown family history of diabetes than that (3.2%) in subjects without a family history of diabetes. Subjects positive for or with an unknown family history of diabetes were older and had a lower level of leisure-time exercise compared to those without a history. Energy intake and the distributions of recruitment groups, gender, smoking habit, and alcohol drinking did not differ between the two groups.

### Associations between serum hs-CRP levels and the prevalence of type 2 diabetes

Table 2 presents the associations between serum hs-CRP level and the prevalence of diabetes. Elevated serum hs-CRP level was proportionally associated with increased prevalence of diabetes after adjusting for probable covariates (*P* for trend was <0.001 in model 2). After additionally adjusting for BMI (model 3), the association remained significant, although it was slightly attenuated (*P* for trend was 0.015). Further adjusting for family history of diabetes did not change the association observed in model 3 (*P* for trend was 0.016 in model 4).

### Combined associations of family history of diabetes and serum hs-CRP level with the prevalence of diabetes

As shown in Table 3, compared to the subjects in the reference group (not having a family history of diabetes and having lower hs-CRP level [≤median]), subjects positive for or with an unknown family history of diabetes and lower hs-CRP level as well as those without a family history and with higher hs-CRP levels (>median) showed significantly high multivariable-adjusted odds ratios of 9.7 (2.6–63.0) and 6.2 (1.7–40.3), respectively, for the prevalence of diabetes (model 3). Having a positive or unknown family history and higher hs-CRP level resulted in a significantly high adjusted odds ratio of 12.5 (3.5–80.0) for the prevalence of diabetes; however, these impacts were not multiplicative, and the interaction effect between family history of diabetes and serum hs-CRP level on the prevalence of diabetes was significant (*P* for interaction was 0.039 in model 3).

### Associations between serum hs-CRP level and the prevalence of diabetes stratified by family history of diabetes

Stratified analyses revealed that elevated serum hs-CRP level was proportionally and significantly associated with an increased prevalence of type 2 diabetes in subjects without a family history of diabetes (*P* for trend was 0.020), but not in those positive for or with an unknown family history nor in those with a family history (Table 4).

## Discussion

The current study utilizing ADTree algorithm, a powerful classification algorithm for data mining (machine learning), and traditional statistical analyses (logistic regression analyses) revealed that elevated serum hs-CRP level, a systemic inflammation biomarker, was proportionally associated with an increased prevalence of type 2 diabetes after adjusting for traditional risk factors including BMI in the Japanese population. The impact of elevated serum hs-CRP level on the prevalence of type 2 diabetes was prominent in subjects without a family history of diabetes.

The prevalence of type 2 diabetes has been rapidly increasing, which has become major worldwide public health and economic problems^27^,^28^. Therefore, it is essential to identify the risk and contributing factors of type 2 diabetes in order to develop preventative measures. Most lifestyle-related diseases, including type 2 diabetes, are multifactorial. Various genetic, lifestyle and environmental risk factors have been identified, including family history due to the similarity in heredity and lifestyles^11^. As presented in the resulting ADTree in the present study, having a positive or unknown family history of diabetes, elevated serum gamma-glutamyl transferase, and suffering from hypertension showed positive impact on the prevalence of type 2 diabetes; these findings are concordant with previous reports^11^,^29^,^30^. Systemic inflammation has also been identified as playing a role in the pathogenesis of various diseases including type 2 diabetes^12^,^13^. CRP is produced by the liver in response to inflammation in the body and is a sensitive systemic biomarker of inflammation^31^. Fibrinogen and hs-CRP are the inflammatory markers most extensively studied for their relation to cardiovascular risk. As for the relation to diabetes, hs-CRP is most frequently studied, and hs-CRP measurement has recently become popular in clinical and health examination settings for assessing low-grade systemic inflammation. Therefore, we used serum hs-CRP as a biomarker of systemic inflammation in the present study. Although a number of studies have reported independent relationships between some inflammatory markers, such as CRP and interleukin (IL)-6, and the risk of developing type 2 diabetes^12^,^13^,^32^,^33^, conclusions about their independent associations have not been consistent between studies. Some studies, including a meta-analysis, have reported no associations after adjusting for adiposity such as BMI or waist circumference and have demonstrated that CRP may not be an independent risk factor for developing type 2 diabetes^34^,^35^.

The combined associations of family history of diabetes and systemic chronic inflammation with the prevalence of type 2 diabetes, investigated by applying a powerful algorithm called ADTree^6^, have been scarcely reported in the Japanese population. Therefore, the present study evaluated the associations of family history of diabetes and serum hs-CRP level with the prevalence of type 2 diabetes in the Japanese population using cross-sectional data. We observed that elevated serum hs-CRP level was significantly and proportionally associated with an increased prevalence of diabetes (*P* for trend was <0.001) after adjusting for probable covariates (model 2). Additional adjustment for BMI (model 3) slightly attenuated this association, but it remained significant. Chronic inflammation level is elevated in obese subjects; serum hs-CRP level was significantly correlated with BMI in our subjects, but this correlation was not so strong (gender-adjusted partial correlation coefficient =0.256, *P* < 0.001) (data not shown). Therefore, we believe that the relationship between elevated serum hs-CRP level and increased prevalence of type 2 diabetes may be explained in part by increased BMI; however, other mechanisms likely contribute to this relationship. Combined analyses (Table 3) revealed that having a positive or unknown family history of diabetes was highly associated with increased prevalence of diabetes in each serum hs-CRP level (≤median and >median) and elevated serum hs-CRP level (>median) was also associated with increased prevalence of diabetes in both subjects without a family history of diabetes and those having a positive or unknown family history. The cut-off value of 0.31 mg/L (median) was nearly equal to that (0.26 mg/L) indicated by ADTree (Fig. 3). Stratified analyses (Table 4) revealed that elevated serum hs-CRP level was proportionally and intensely associated with an increased prevalence of diabetes among subjects without a family history of diabetes. Although having a family history and elevated systemic inflammation are independently associated with the prevalence of diabetes each other, the impact of systemic inflammation on diabetes might be obvious in subjects without a family history of diabetes.

The mechanisms underlying the relationships between elevated serum hs-CRP level and increased prevalence of type 2 diabetes cannot be entirely understood, however, there are several plausible mechanisms. Human CRP plays an active role in inducing hepatic insulin resistance in rats, partially by activating extracellular signal-regulated kinase (ERK), with downstream impairment in the insulin signaling pathway^36^. Tumor necrosis factor alpha (TNF-α) and interleukin-6 (IL-6), which are pro-inflammatory cytokines secreted by adipose tissue, can stimulate CRP production in the liver^37^. TNF-α is also known to induce insulin resistance^38^. A mouse study reported that chronic exposure to IL-6 inhibits insulin receptor signal transduction in primary hepatocytes^39^. The relationship between elevated serum hs-CRP level and glucose metabolism disorders may be intermediated by increased secretion of TNF-α and IL-6. Further studies enhancing and attenuating CRP function or production are necessary to determine the causal effects of CRP on glucose metabolism.

This study has several limitations. First, because of the cross-sectional study design, the causal relationship between serum hs-CRP and the prevalence of type 2 diabetes should be interpreted with caution. Second, although the analyses adjusted for a number of potential confounding factors, residual confounding by unmeasured genetic, lifestyle, or environmental factors cannot be eliminated. Third, information about family history of diabetes and other lifestyle factors was self-reported; therefore, non-differential misclassification may have been inevitable. Finally, since all of our subjects were Japanese, our results may not be generalizable in other ethnic populations. Despite these limitations, we consider our results to be meaningful for the prevention of type 2 diabetes. Measuring hs-CRP level is now convenient and popular in health examination and may be useful for the identification of individuals in the Japanese population at high risk of type 2 diabetes, especially those without a family history of diabetes.

In conclusion, our study demonstrates that systemic inflammation as measured by serum hs-CRP was proportionally associated with an increased prevalence of type 2 diabetes after adjusting for BMI in the Japanese population, especially in subjects without a family history of diabetes. Further larger and prospective studies are necessary to confirm these associations and causality between serum hs-CRP level and type 2 diabetes.

## Additional Information

**How to cite this article:** Uemura, H. *et al*. Systemic inflammation and family history in relation to the prevalence of type 2 diabetes based on an alternating decision tree. *Sci. Rep.* **7**, 45502; doi: 10.1038/srep45502 (2017).

**Publisher's note:** Springer Nature remains neutral with regard to jurisdictional claims in published maps and institutional affiliations.

## Figures and Tables

**Table 1:** Clinical characteristics of the subjects according to their family history of diabetes.

	Family history of diabetes		
	Negative	Positive/Unknown	*P*
	(n = 626)	(n = 476)	
Gender^c^			
Men	319 (51.0)	237 (49.8)	0.701
Women	307 (49.0)	239 (50.2)	
Recruit group^c^			
Health Check-up Center	295 (47.1)	218 (45.8)	0.662
Participants by leaflet	331 (52.9)	258 (54.2)	
Age (years)^a^	52.0 ± 9.9	53.6 ± 9.5	0.009
BMI (kg/m^2^)^b^	22.8 (21.0, 25.2)	23.1 (21.0, 25.8)	0.078
Smoking status^c^			
Current	94 (15.0)	83 (17.4)	0.521
Past	162 (25.9)	124 (26.1)	
Never	370 (59.1)	269 (56.5)	
Alcohol drinking^c^			
Current	345 (55.1)	261 (54.8)	0.716
Past	8 (1.3)	9 (1.9)	
Never	273 (43.6)	206 (43.3)	
Leisure-time exercise (MET-hours/week)^b^	7.65 (1.28, 23.10)	5.10 (1.28, 17.85)	0.046
Carbohydrate intake (g/day)^b^	234 (198, 278)	233 (195, 274)	0.712
Energy intake (kcal/day)^b^	1677 (1490, 1904)	1660 (1471, 1862)	0.274
Serum hs-CRP (mg/L)^b^	0.30 (0.15, 0.61)	0.32 (0.16, 0.70)	0.316
Fasting plasma glucose (mg/dL)^b^	92 (87, 99)	93 (88, 101)	0.022
Diabetes prevalence^c^	20 (3.2)	42 (8.8)	<0.001

^a^Mean ± SD, ^b^Median (25%, 75%), ^c^Number (%).

BMI, body mass index; MET, metabolic equivalent; hs-CRP, high-sensitivity C-reactive protein.

Differences are analyzed by *t*-test ^a^, Wilcoxon rank sum test ^b^, or chi-square test ^c^.

**Table 2:** Associations between serum hs-CRP and the prevalence of type 2 diabetes.

	Serum hs-CRP			*P for trend*
	Q1 + Q2 (≤0.31 mg/L)	Q3 (>0.31∼0.66 mg/L)	Q4 (>0.66 mg/L)	
	OR (95% CI)	OR (95% CI)	OR (95% CI)	
Prevalence rate of diabetes (%)	3.0	6.4	10.2	
Model 1	1	1.7 (0.86, 3.5)	3.0 (1.6, 5.7)	<0.001
Model 2	1	1.9 (0.91, 3.8)	3.2 (1.7, 6.3)	<0.001
Model 3	1	1.5 (0.68, 3.1)	2.4 (1.2, 4.8)	0.015
Model 4	1	1.5 (0.71, 3.3)	2.4 (1.2, 4.9)	0.016

hs-CRP, high-sensitivity C-reactive protein; Q2, first quartile; Q3, second quartile; Q4, third quartile; Q1, fourth quartile.

OR, odds ratio; CI, confidence interval.

Model 1: adjusted for age and sex.

Model 2: adjusted for age, sex, recruit group, smoking status, current alcohol drinkings, leisure-time exercise, carbohydrate intake, and energy intake.

Model 3: adjusted for the covariates in model 2 plus body mass index.

Model 4: adjusted for the covariates in model 3 plus family history of diabetes.

**Table 3:** Combined associations of family history of diabetes and serum hs-CRP with the prevalence of type 2 diabetes.

Serum hs-CRP	Family history of diabetes			
	Negative		Positive/Unknown	
	OR	(95% CI)	OR	(95% CI)
Model 1				
≤Median	1		10.1	(2.8–64.6)
>Median	8.2	(2.3–51.8)	15.5	(4.5–97.4)
*P_interaction_	0.023			
Model 2				
≤Median	1		9.8	(2.7–63.2)
>Median	8.5	(2.4–54.1)	17.2	(5.0–108.5)
*P_interaction_	0.035			
Model 3				
≤Median	1		9.7	(2.6–63.0)
>Median	6.2	(1.7–40.3)	12.5	(3.5–80.0)
*P_interaction_	0.039			

hs-CRP, high-sensitivity C-reactive protein; OR, odds ratio; CI, confidence interval.

The median value of hs-CRP was 0.31 mg/L.

Model 1: adjusted for age and sex.

Model 2: adjusted for age, sex, recruit group, smoking status, current alcohol drinking, leisure-time exercise, carbohydrate intake, and energy intake.

Model 3: adjusted for the covariates in model 2 plus body mass index.

*P values for interaction of family history of diabetes (no, yes) and serum hs-CRP (≤median, > median).

**Table 4:** Associations of serum hs-CRP with the prevalence of diabetes stratified by family history of diabetes.

Serum hs-CRP	Family history of diabetes					
	Negative (n = 626)		Positive/Unknown (n = 476)		Positive only (n = 271)	
	OR	(95% CI)	OR	(95% CI)	OR	(95% CI)
Model 1						
Q1 + Q2	1		1		1	
Q3	7.3	(1.8–48.9)	1.0	(0.40–2.4)	1.2	(0.33–4.1)
Q4	10.2	(2.6–67.2)	2.0	(0.92–4.2)	1.5	(0.56–4.3)
*P for trend*	0.002		0.086		0.401	
Model 2						
Q1 + Q2	1		1		1	
Q3	7.5	(1.8–50.8)	1.1	(0.44–2.8)	1.1	(0.28–4.0)
Q4	12.1	(3.0–80.6)	2.2	(0.99–4.8)	1.7	(0.55–5.1)
*P for trend*	<0.001		0.056		0.372	
Model 3						
Q1 + Q2	1		1		1	
Q3	6.0	(1.4–42.5)	0.84	(0.30–2.2)	0.85	(0.18–3.6)
Q4	7.1	(1.6–50.0)	1.7	(0.70–4.0)	1.3	(0.36–5.0)
*P for trend*	0.020		0.218		0.632	

hs-CRP, high-sensitivity C-reactive protein; Q1, first quartile; Q2, second quartile; Q3, third quartile; Q4, fourth quartile.

OR, odds ratio; CI, confidence interval.

Model 1: adjusted for age and sex.

Model 2: adjusted for age, sex, recruit group, smoking status, current alcohol drinking, leisure-time exercise, carbohydrate intake, and energy intake.

Model 3: adjusted for the covariates in model 2 plus body mass index.
